# Alpha-2 Agonists in Children and Adolescents With Post-traumatic Stress Disorder: A Systematic Review

**DOI:** 10.7759/cureus.53009

**Published:** 2024-01-26

**Authors:** Amit Jagtiani, Raghu Gandhi, Akshat Banga, Jacquetta Blacker, Riecha Joshi, Bhaswanth Bollu, Rahul Kashyap

**Affiliations:** 1 Psychiatry, Burrell Behavioral Health, Springfield, USA; 2 Psychiatry, Abbott Northwestern Hospital, Minneapolis, USA; 3 Internal Medicine, Sawai Man Singh Medical College, Jaipur, IND; 4 Psychiatry and Behavioral Sciences, University of Minnesota, Minneapolis, USA; 5 Pediatrics, Government Medical College, Kota, Kota, IND; 6 Emergency Medicine, All India Institute of Medical Sciences, New Delhi, IND; 7 Medicine, Drexel University College of Medicine, Philadelphia, USA; 8 Global Clinical Scholars Research Training (GCSRT), Harvard Medical School, Boston, USA; 9 Research, Global Remote Research Program, Saint Paul, USA; 10 Critical Care Medicine, Mayo Clinic, Rochester, USA; 11 Research, WellSpan Health, York, USA

**Keywords:** guanfacine, clonidine, α2 agonists, adolescents, children, post-traumatic stress disorder

## Abstract

Exposure to traumatic stress is common among children. Post-traumatic stress disorder (PTSD) is a debilitating chronic mental disorder that can develop following exposure to a traumatic event. Psychopharmacological research in pediatric PTSD is limited. There is some evidence supporting the use of alpha-2 (α_2_) agonists for symptoms associated with PTSD. This systematic review identified published studies evaluating the effectiveness of α_2_ agonists in treating PTSD symptoms in children and adolescents. We conducted an extensive literature search on PubMed, MEDLINE, EMBASE, Cochrane Collaboration, and PsycINFO databases for published articles that evaluated the use of α_2 _agonists (clonidine and guanfacine) for treating symptoms of PTSD in children and adolescents. The study protocol was registered in Prospero (ID: CRD42021273692) and followed the PRISMA guidelines. A total of 10 published articles about clonidine or guanfacine use in PTSD in children and adolescents were identified. Studies found clonidine effective in reducing PTSD symptoms; however, the effects were variable. Clonidine and guanfacine showed effectiveness in treating nightmares, hyperarousal, aggression, and sleep disturbances and reducing re-experiencing, avoidant, and hyperarousal symptom clusters. No randomized, double-blind, placebo-controlled trials were found during the literature search. α_2_ agonists’ effectiveness in treating symptoms associated with PTSD in children and adolescents is preliminary. Future placebo-controlled trials are needed to assess the efficacy and safety of α_2_ agonists.

## Introduction and background

Post-traumatic stress disorder (PTSD) is a debilitating chronic mental disorder that can develop following exposure to a traumatic event. Children are more susceptible to developing reactive, exaggerated, or avoidant responses to traumatic stressors because they lack the ability to form effective coping mechanisms [1]. Approximately 60% of children in the United States are exposed to a potentially traumatic experience at least once in their lifetime [2]. One of the forms of trauma is abuse (physical, sexual, or emotional). The most common forms of abuse reported in children are physical (10-34%) and sexual (10%) [3]. Even bullying can result in significant psychological consequences, including PTSD [4]. Although overall rates of trauma exposure are similar in boys and girls, boys are more likely to suffer physical violence, whereas sexual violence is more likely in girls [2,3]. Epidemiological studies in high-income countries have suggested that around 10% of youth exposed to trauma develop PTSD [2].

According to the Diagnostic and Statistical Manual of Mental Disorders (DSM-5), PTSD is characterized by collective symptoms persisting for more than one month after a traumatic event. These include intrusive symptoms (recurrent flashbacks, nightmares, re-enacting the traumatic event), avoidance (avoidance of thoughts, conversations, and reminders of the trauma, anhedonia, social isolation), and numbing, increased arousal (hypervigilance, exaggerated startle response, insomnia, aggressive behavior), and mood instability. In children, trauma-specific re-enactment can happen in play, and for children younger than six years, there might be frightening dreams without recognizable content [5]. PTSD often co-occurs with depression, anxiety, and attention deficits that further impair the daily functioning of children [6].

Management strategies for pediatric PTSD currently include trauma-focused cognitive behavior therapy (TF-CBT), eye movement desensitization and reprocessing (EMDR), and psychopharmacology [7]. Even though TF-CBT is the best evidenced first-line treatment modality for PTSD, it has its limitations due to the paucity of trained psychotherapists, considerable treatment drop-out and non-response rates, and ineffectiveness in patients suffering from ongoing trauma or living in socially insecure circumstances that make its application challenging [8-10]. Comparably, in contrast to adult literature, the research-based evidence for the pharmacological treatment of pediatric PTSD is also limited. Though selective serotonin reuptake inhibitors (SSRIs) like sertraline and paroxetine and alpha-1 antagonists like prazosin have been approved by the Food and Drug Administration (FDA) for treatment in adults, these drugs are not approved for PTSD in the pediatric age group, and thus there are no specific medications approved for children and adolescents with PTSD [11-13]. However, SSRIs are commonly used in clinical practice for PTSD in children and adolescents when therapy is not effective.

Recent studies have found some evidence supporting the use of alpha-2 (α_2_) agonists (clonidine and guanfacine) for managing symptoms associated with PTSD [14,15]. Studies have demonstrated a correlation between stress-induced catecholamine surge in the prefrontal cortex (PFC) and alterations in brain physiology that may partially explain the pathophysiological and neuroendocrine mechanisms of PTSD [16,17]. The increased norepinephrine (NE) levels create a hyper-noradrenergic state which has been linked to symptoms such as hypervigilance, exaggerated startle response, and sleep disruption [18]. By contrast, activating the α_2_ receptor decreases the activity of presynaptic calcium channels leading to a decrease in the NE levels [19,20]. The α_2_ agonists act on these receptors to strengthen prefrontal cortical NE connectivity, thus improving behavioral inhibition, working memory, and impulse control [18,21,22].

Although clonidine and guanfacine have proven efficacy in treating attention deficit hyperactivity disorder (ADHD), anxiety disorders, and sleep disorders, evidence of their utility in pediatric PTSD is limited. There have been previous reviews and meta-analyses on the pharmacological treatment of PTSD in children and adolescents [23-25]; however, the application of α_2_ agonists remains yet to be addressed. The present study aims to provide a comprehensive assessment of the current knowledge on the efficacy of α_2_ agonists in pediatric PTSD and discuss the implications of our findings to direct future research within the field.

## Review

Methods

We conducted a comprehensive literature search of human literature via PubMed, MEDLINE, EMBASE, Cochrane Collaboration, and PsychINFO of all dates up to April 20th, 2023. The search string was title/MeSH terms/full text (“alpha (2 receptor) agonist” OR “adrenergic (alpha 2 receptor) agonist*” OR “clonidine” OR “clonidine hydrochloride” OR “guanfacine” OR “guanfacine hydrochloride”) AND (“PTSD” OR “posttraumatic stress” OR “hyperarousal” OR “irritability” OR “anger outburst” OR “temper outburst” OR “reckless behavior*” OR “impulsive*” OR “sleep disturbance” OR “hypervigilance” OR “startle (reaction*)” OR “arousal disorder, sleep” OR irritable mood” OR “anger” OR “behavior(s), impulsive” OR “aggression(s)” OR “hypervigilance”) AND (“children” OR “adolescents” OR “pediatric”). Articles were collated in Endnote 20 (Clarivate Analytics, Philadelphia, PA). Duplicates were removed manually by the first author. Each abstract was reviewed independently by the first and second authors.

**Figure 1:**
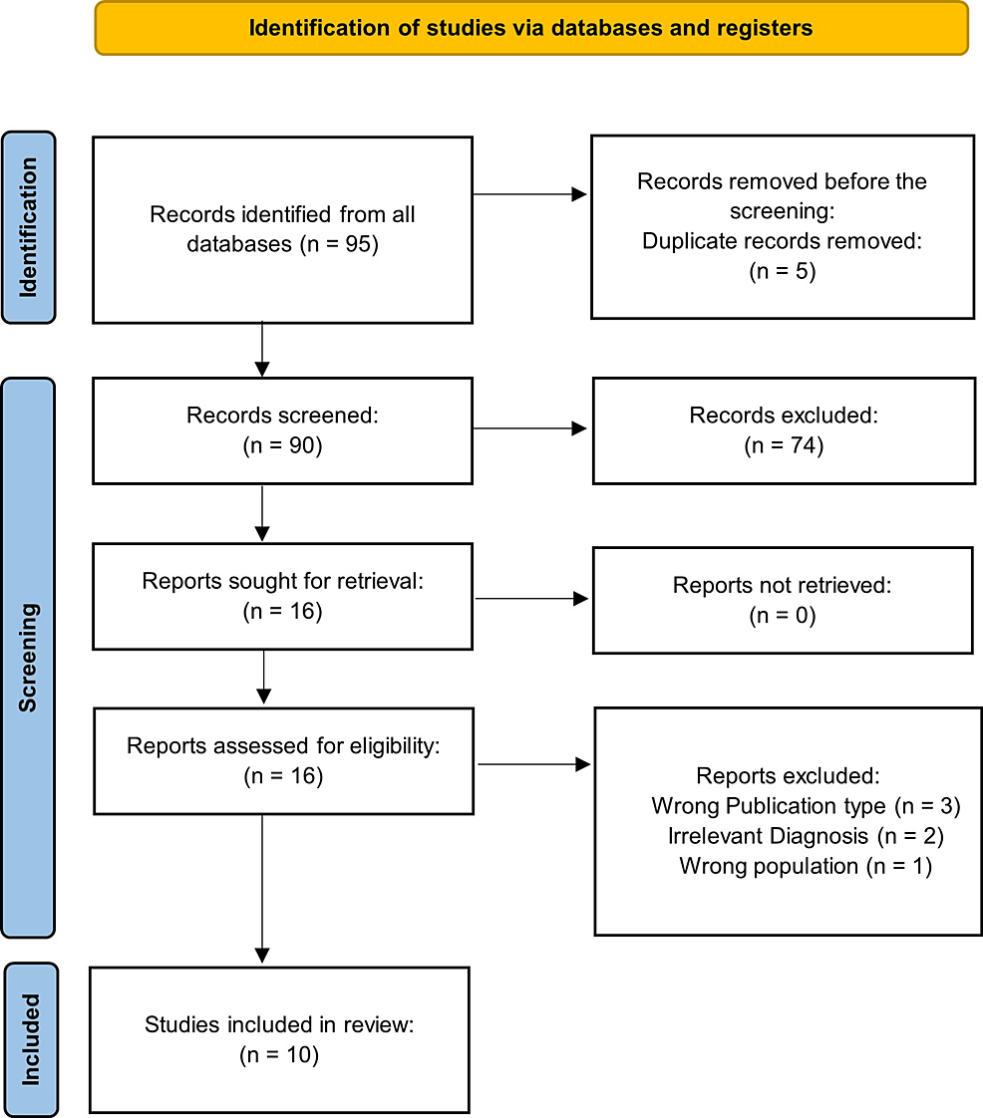
PRISMA flowchart outlining the study search PRISMA: Preferred Reporting Items for Systematic Reviews and Meta-Analyses

The study protocol was registered in Prospero (ID: CRD42021273692) and followed the PRISMA (Preferred Reporting Items for Systematic Reviews and Meta-Analyses) guidelines. The study was registered. Inclusion criteria were: (1) human subjects aged up to 18 years treated for PTSD or associated symptoms with the alpha-agonists clonidine and/or guanfacine; (2) qualitative or quantitative outcomes of the intervention were reported in primary studies; and (3) full-text was available in English. Exclusion criteria were: (1) no diagnosis of PTSD; and (2) non-primary literature, i.e., review articles. Ninety-five studies were identified. Five duplicates were removed. Ninety abstracts were screened for relevance by two authors (Figure 1). Seventy-four articles were excluded for diagnoses other than PTSD, review articles, non-pediatric population, and non-relevant/unspecified pharmacological intervention. Conflicts of uncertain studies were determined by discussion or review of the article’s full text to make a definitive judgment. A total of 16 articles were assessed for eligibility via full-text analysis. Finally, 10 studies met the full inclusion criteria. Data were extracted independently and in duplicate by all authors.

Data on the nature of the study, study population characteristics (age and sex/gender), drug choice (clonidine and/or guanfacine), drug dosage, impact on symptoms associated with PTSD, and associated comorbid conditions were extracted. The effect of α_2_ agonists on PTSD-associated symptoms was the study's primary outcome. Secondary outcomes were the presence of associated comorbidities and drug-related adverse effects.

The National Institutes of Health (NIH) Scale was used to grade all included studies for the strength of evidence of case reports and open-label trial-based studies [26]. Studies were classified into three categories based on the scale: good, fair, or poor. Two authors (AB and RJ) independently performed the quality assessment of the included studies; any discrepancies were resolved through discussion.

Results

A total of 16 published articles describing the use of α_2_ agonist drugs in children and adolescents with PTSD were identified and assessed for eligibility. Out of the 16 articles, six papers were further excluded due to irrelevant (not PTSD) diagnosis (N=2), irrelevant publication type (N=3), and irrelevant study population (N=1). Ultimately, ten studies consisting of three open-label trials and seven case reports met the inclusion criteria. No randomized, double-blinded controlled trials were found in our literature search. An overview of the included studies is provided in Table 1. Four studies used guanfacine [27-30], five used clonidine [31-35], and a sole study took both clonidine and guanfacine into consideration consecutively [36].

**Table 1:** Study characteristics of included research studies SACRED: Screen for Child Anxiety Related Emotional Disorders; GAD: Generalized anxiety disorder; CIS: Columbia Impairment Scale; GXR: Guanfacine extended-release; ADHD: Attention-deficit/hyperactivity disorder; UCLA: University of California at Los Angeles; PTSD: Post-traumatic stress disorder; CBT: Cognitive behavioral therapy; BDI: Beck Depression Inventory; BAI: Beck Anxiety Inventory; MPH: Methylphenidate; NAA+NAAG: N-acetylaspartate+N-acetylaspartylglutamate

Study Name	Study Type	No. of Participants	Age/Sex/Setting	Medication (Dose)	Outcome	Adverse Events	Risk of Bias/Comments
Anderson et al., 2020 [27]	Case report	1	15/female/inpatient	Guanfacine dose: 1 mg qhs followed by 2 mg qhs	Improvement in intrusive thoughts, impulsive self-injurious behavior, suicidal ideation, hypervigilance, avoidance symptoms, mood, volitional conduct, sleep symptoms	Dizziness, psychomotor slowing	Comorbidity: major depressive disorder, cannabis use disorder, alcohol use disorder, primary nocturnal enuresis, insomnia. Cointerventions: dialectical behavioral therapy. At initial admission: aripiprazole 2 mg qhs and fluoxetine 60 mg qam, trazodone 50 mg qhs prn. At readmission: fluoxetine 60 mg qam, bupropion XL 150 mg qam. Positive challenge/rechallenge
Connor et al., 2013 [28]	Open-label study	19	6 to 18/male and female/outpatient	Guanfacine ER dose range: 1 to 4 mg; mean dose: 1.9 mg ± 0.35 mg	Significant improvement in symptoms of reexperiencing, avoidant, hyperarousal. Outcome measures: UCLA PTSD Reaction Index (parent-rated), SCARED-GAD subscale, CIS, ADHD-RS-IV	Dry mouth was a commonly reported on-drug side effect. One patient dropped out of the study, citing worsening depression on GXR.	19 children, 8 weeks open-label study. Comorbidity: ADHD (89.5%), PTSD (68.4%), GAD (47.4%), depression (21.1%), separation anxiety disorder (10.6%), and reactive attachment disorder (5.3%). Cointerventions: psychotherapy (7 patients). No stratification of results based on diagnosis and severity of symptoms. Compliance monitored. Dropouts not accounted
Lyon et al., 2008 [29]	Case report	1	7/female/outpatient	Clonidine 0.05 mg at bedtime for 3 weeks, followed by guanfacine 0.5 mg at bedtime for 7 weeks	Nightmares resolved on clonidine and guanfacine. Other PTSD symptoms not studied. No rating scales	Not described	Breakthrough nightmares in the early morning on clonidine, which resolved with a switch to guanfacine. Cointerventions: unclear. Possible ongoing trauma therapy
Wu et al., 2020 [30]	Case report	1	17/female/emergency room (ER) followed by inpatient	Guanfacine ER dose: 1 mg qhs on day 3, increased to 2 mg qhs by day 7	Improvement in nightmares, self-harm, suicidal ideation, hyperarousal symptoms, intrusive thoughts/flashbacks/images, sleep	Not described	Comorbidity: major depressive disorder, self-mutilation, cannabis use disorder, alcohol use disorder, unspeciﬁed eating disorder (resolved), postural orthostatic tachycardia syndrome, childhood-onset asthma, migraines. Cointerventions: psychotherapy, sumatriptan, sertraline 25 mg daily, hydroxyzine 25 mg daily, trazodone 50 mg qhs, mirtazapine 7.5 mg prn
De Bellis et al., 2001 [31]	Case report	1	11/female/residential treatment	Clonidine dose: 0.1 mg three times a day	Improvement in hyperarousal, nightmares, sexual re-enactment, aggression, and mood symptoms. Discontinuation of clonidine led to reemergence of symptoms, with resolution on restarting. Sustained improvement on returning home from residential treatment	Not described	Cointerventions: counseling, milieu therapy (residential). No rating scales
Harmon et al., 1996 [32]	Open-label study	7	3 to 6/male and female/preschool day program	Clonidine dose range: 0.1 to 0.2 mg per day	Significant improvement in symptoms of aggression (n=7), impulsivity (n=5), hyperarousal (n=5), hypervigilance (n=5), anxiety (n=5), oppositionality (n=5), insomnia and nightmares (n=5). No rating scales	Most patients experienced sedation. No other major side effects	Comorbidity: depressive features, attentional problems, and reactive attachment disorder. Cointerventions: individual, family, milieu therapy, behavioral interventions, imipramine (2 patients)
Perry et al., 1994 [33]	Open-label study	17	6 to 14/males and females/outpatient	Clonidine dose: 0.05 mg bid followed by 0.1 mg bid for a total of 4 weeks	Improvement in behavioral impulsivity, anxiety, arousal, concentration, mood, pre-psychotic and psychotic symptoms	Sedation	17 children, 4 weeks open-label study. Used PSAS system for symptom measurement. Treatment progress assessed by teachers, childcare workers, and therapists
Porter et al., 1999 [34]	Case report	1	12/male/residential treatment	Guanfacine dose: 2 mg twice daily	Improvement in residual irritability and impulsivity (after MPH optimization). Outcome measures: Number of crisis interventions, manual restraints, and stat meds	Not described	Comorbidity: ADHD and reactive attachment disorder. Cointerventions: play therapy, CBT, pet therapy, family therapy, milieu therapy (inpatient). Medications: concerta 54 mg, sertraline 125 mg daily
Ye et al., 2019 [35]	Case report	1	7/male/outpatient	Clonidine dose: 0.05 mg at bedtime for 2 weeks, followed by 0.1 mg at bedtime	Improvement in intrusion, insomnia, nightmares, hypervigilance, irritability, avoidance symptoms. Sustained over 6 months of treatment. No rating scales	Not described	Improvement in medial prefrontal cortex NAA+NAAG/creatine concentrations on clonidine treatment. Patient received supportive/cognitive psychotherapy during the treatment. Positive challenge/rechallenge
Horrigan et al., 1996 [36]	Case report	1	18/male/outpatient	Transdermal clonidine dose: 0.2 mg per day	Improvement in agitation, insomnia, anxiety, and depressive symptoms. Outcome measures: BDI and BAI	Not described	Comorbidity: depression, anxiety, ADHD, and learning disorder. Cointerventions: aripiprazole 5 mg, fluvoxamine 250 mg, and lamotrigine 200 mg

Perry *et al. *conducted the first open-label study in 1994 investigating the use of α_2_ agonists in PTSD in children and adolescents with a focus on cardiovascular changes in PTSD [33]. They reported a decrease in arousal symptoms and improvement in behavioral impulsivity, anxiety, arousal, and mood, along with a reduction in basal heart rate after four weeks. In an open-label trial [36], clonidine was tried on seven preschool children and was found to be highly effective in reducing PTSD symptoms. All seven children showed improvement in aggressive behavior and interpersonal relationships with peers and their teachers. Researchers also observed recovery from symptoms including impulsivity, emotional outbursts, mood lability, hyperarousal, hypervigilance, generalized anxiety, oppositionality, insomnia, and nightmares in five of seven children. Another open-label trial using guanfacine extended-release (GXR) hypothesized that guanfacine might reduce intrusive and hyperarousal symptoms in children [28]. Their study showed improvement in the University of California at Los Angeles (UCLA) PTSD Reaction Index, with more than two-thirds of participants stating a reduction of re-experiencing, avoidant, and hyperarousal symptoms when treated with 1 to 4  mg of guanfacine at bedtime. To an extent, the study also showed promising results in controlling sleep difficulties, nightmares, and associated anxiety [28].

Horrigan *et al. *published the first case report on the use of α_2_ agonist in a pediatric PTSD patient in 1996 [36]. They used clonidine followed by guanfacine for the focused treatment of traumatic stress-related nightmares and found the drugs to be effective in alleviating symptoms. In 1999, Porter *et al.* assessed the tolerability and efficacy of clonidine in a victim of sexual molestation [34]. Clonidine was well tolerated and successfully reduced hyperarousal symptoms, perception of distorted reality, disorganized thinking, aggressive behavior, nightmares, and mood changes. A case report by De Bellis *et al.* in 2001 established an association between clonidine use and the initial resolution of PTSD symptoms, including insomnia, intrusion, nightmares, hypervigilance, and irritability [31]. Relapse of symptoms after discontinuing the α_2_ agonist drug further implicated its role in controlling PTSD symptoms in the patient. Lyon *et al.*, in 2008, used guanfacine to control impulsivity and irritability associated with PTSD effectively [29]. Their study showed additional benefits in controlling hyperactive, aggressive behavior. The transdermal patch form of clonidine was used by Ye *et al.* to reflect its usefulness in managing agitation, insomnia, hyperarousal, and trauma-related flashbacks while also elevating mood and suppressing suicidal ideation in an adolescent who had PTSD [35]. Her symptoms, however, returned rapidly on discontinuation of clonidine. In another case report, guanfacine successfully suppressed intrusive thoughts, impulsive self-injurious behavior, and suicidal ideation, controlled the patient’s hypervigilant and avoidant symptoms, and improved her mood, volitional conduct, and sleep symptoms. Discontinuation of the α_2_ agonist led to quick relapse and recurrence of PTSD symptoms in the patient [27]. In a recent case report by Wu *et al.*, an adolescent female showed a sustained enhancement in her mood, resolution of intrusive flashbacks and images, and resolution of nightmares leading to improvement in sleep on guanfacine [30].

Amongst all the above studies using clonidine, the dosage of clonidine ranged between 0.05 and 0.3 mg per day, whereas the dose of guanfacine ranged between 0.5 and 4 mg per day among the included studies focusing on guanfacine use in pediatric PTSD patients.

The quality assessment of the included studies based on the NIH Scale is given in Tables 2-3 [26]. For the non-blinded, open-label trial studies [28,32,33], quality assessment of the three included studies identified all three as having a poor quality of evidence. The remaining seven studies were all case reports [27,29-31,34-36]. Most of the case reports were graded as fair-quality studies based on the NIH Scale.

**Table 2:** Risk of bias for case report studies based on the NIH Scale 1: Yes; 0: No; NA: Not applicable; NIH: National Institutes of Health

Case Reports	1. Was the study question or objective clearly stated?	2. Was the study population clearly and fully described, including a case definition?	3. Were the cases consecutive?	4. Were the subjects comparable?	5. Was the intervention clearly described?	6. Were the outcome measures clearly defined, valid, reliable, and implemented consistently across all study participants?	7. Was the length of follow-up adequate?	8. Were the statistical methods well-described?	9. Were the results well-described?	Quality Rating (Good, Fair, or Poor)
Anderson et al., 2020 [27]	1	1	NA	NA	1	0	1	NA	1	Fair
Lyon et al., 2008 [29]	1	1	NA	NA	1	1	1	NA	1	Fair
Wu et al., 2020 [30]	1	1	NA	NA	0	0	0	NA	1	Poor
De Bellis et al., 2001 [31]	1	0	NA	NA	1	0	1	NA	1	Fair
Porter et al., 1999 [34]	1	1	NA	NA	1	0	1	NA	1	Fair
Ye et al., 2019 [35]	1	1	NA	NA	1	0	1	NA	1	Fair
Horrigan et al., 1996 [36]	1	0	NA	NA	0	0	1	NA	0	Poor

**Table 3:** Risk of bias for open-label trial studies based on the NIH Scale 1: Yes; 0=No; NA: Not applicable; NIH: NIH: National Institutes of Health

Open-Label Studies	1. Was the study described as randomized, a randomized trial, a randomized clinical trial, or an RCT?	2. Was the method of randomization adequate (i.e., use of randomly generated assignment)?	3. Was the treatment allocation concealed (so that assignments could not be predicted)?	4. Were study participants and providers blinded to treatment group assignment?	5. Were the people assessing the outcomes blinded to the participants' group assignments?	6. Were the groups similar at baseline on important characteristics that could affect outcomes (e.g., demographics, risk factors, co-morbid conditions)?	7. Was the overall drop-out rate from the study at endpoint 20% or lower of the number allocated to treatment?	8. Was the differential drop-out rate (between treatment groups) at endpoint 15 percentage points or lower?	9. Was there high adherence to the intervention protocols for each treatment group?	10. Were other interventions avoided or similar in the groups (e.g., similar background treatments)?	11. Were outcomes assessed using valid and reliable measures implemented consistently across all study participants?	12. Did the authors report that the sample size was sufficiently large to be able to detect a difference in the main outcome between groups with at least 80% power?	13. Were outcomes reported or subgroups analyzed prespecified (i.e., identified before analyses were conducted)?	14. Were all randomized participants analyzed in the group to which they were originally assigned, i.e., did they use an intention-to-treat analysis?	Quality Rating (Good, Fair, or Poor)
Connor et al., 2013 [28]	0	0	0	0	0	NA	NA	NA	1	0	1	0	1	NA	Poor
Harmon et al., 1996 [32]	0	0	0	0	0	NA	NA	NA	1	0	0	0	1	NA	Poor
Perry et al., 1994 [33]	0	0	0	0	0	NA	NA	NA	1	NA	1	0	1	NA	Poor

Discussion

The use of pharmacotherapy for PTSD in children and adolescents is common due to the ability to improve daily functioning in life by controlling disabling symptoms and reducing emotional distress, thus diminishing functional impairment [33]. The studies included in this systematic review showed that α_2_ agonists were used in children and adolescents demonstrating extreme emotional dysregulation and increased arousal (hypervigilance, exaggerated startle response, insomnia, aggressive behavior). α_2_ agonists were used as a bridge to allow the slower process of psychotherapy to begin working or as a substitute when psychotherapy was inaccessible or intolerable for the patient.

This systematic review evaluates the efficacy of α_2_ agonists in children and adolescents with PTSD. Our study found clonidine and guanfacine to be effective in controlling symptoms of sympathetic overactivity, including but not limited to nightmares and insomnia, hyperarousal, intrusive thinking, avoidance, and aggressive/irritable behavior commonly associated with PTSD. We also found a recurrence of PTSD symptoms after discontinuing the α_2_ agonist following the initial resolution of these symptoms [27,31,34,35]. Attention disorders (ADHD) and depressive symptoms were the most common comorbidities in the included studies, followed by anxiety disorders and reactive attachment disorder. Patients on α_2_ agonists usually reported dryness of mouth, increased sedation, and dizziness as common adverse effects of the treatment [27,28,33,36]. According to Sallee *et al.* (2009) [37], the recommended doses for the management of ADHD of clonidine and guanfacine are 0.1-0.4 mg/d and 1-4 mg/d, respectively. The dosing range used for the management of PTSD in the cases reported in this study is comparable to the recommended doses for ADHD.

Comparison With Existing Literature

There are multiple studies and more significant data on the effectiveness of α_2_ agonists in treating specific symptoms seen in PTSD in adults when compared to the pediatric database. The findings in placebo-controlled trials by Neylan *et al.* and Davis *et al.* showed no significant improvement in PTSD symptoms with the use of α_2_ agonist guanfacine [38,39]. However, the use of clonidine in treating sleep abnormalities is well documented. Kinzie *et al.* reported improvement in PTSD symptoms [40,41], including decreased insomnia and a reduction in nightmare frequency in two case series of Cambodian refugees with PTSD and comorbid depression. A retrospective review of 27 individual prescription regimens or trials in adults by Detweiler *et al.* found clonidine to be effective in reducing the frequency and severity of nightmares in 63% of trials, but a lack of statistical analysis and comparison of unmatched patient data amongst different studies due to a lack of randomization limited the scope of their results [42]. Case reports by Bange and Melvin and Alao *et al. *showed clonidine to effectively control nightmare symptoms in war veterans [20,21]. Bange and Melvin reported a return of nightmare symptoms when the female veteran was taken off clonidine. Her nightmares again resolved once clonidine was resumed [20].

Evidence from case reports and clinical trials indicates that guanfacine can be helpful in boosting top-down control to reduce impulsive, self-injurious, and repetitive behaviors in children. Multiple clinical trials, including Hunt *et al.* and Hervas *et al. *have found α_2_ agonists to be effective in improving impulse control in children and adolescents with ADHD [43,44]. Comparatively, Propper *et al. *and Politte *et al.* also reported improvement in self-injurious and repetitive behavior in autistic children with guanfacine [45,46]. Philipsen *et al.* found clonidine to be effective in controlling acute states of aversive inner tension and the urge to commit self-injurious behaviors in adult female patients with bipolar disorders [47]. These results are supported by our systematic review, which showed improvement in self-injurious, aggressive behavior and impulse control across six studies in child and adolescent PTSD patients [27,29,30,32-34]. Also, most of these studies included in our review had significant comorbidity with ADHD, depression, and anxiety, which could explain the benefits of alpha-agonists.

About two studies in our systematic review demonstrated sedation as a significant side effect [32,33]. Clonidine caused initial moderate sedation during its first week of use, which was noted to be transient; however, in the same study, using a clonidine patch was associated with lesser initial sedation [32]. In a rare case, one patient developed severe post-streptococcal glomerulonephritis with hypertension during treatment with clonidine [32], while in another study, a patient reported worsening comorbid depression with clonidine [28]. Geyskes *et al.* studied 14 patients with essential hypertension treated with clonidine for more than one month. They showed a rebound rise of blood pressure on clonidine withdrawal, explained by overactivity of the sympathetic nervous system [48]. Connor *et al.* detailed that worsening depression could be a side effect of ongoing clonidine treatment [49]. According to a review of α_2 _agonists by Yasaei *et al.*, depression is a rare but plausible side effect of α_2_ agonist use [50].

Possible Mechanisms Underlying the Findings

The exact underlying mechanism of the development of PTSD following trauma yet remains to be fully understood. However, disruption of the autonomic nervous system and sympathetic response secondary to alterations in basal NE level, along with the excessive release of NE and symptoms related to this hyper-noradrenergic state following a traumatic event or its reminders, have been shown to play a role in the pathophysiology of PTSD [51,52]. Psychoneurological evaluation of trauma victims and neuroimaging evidence have shown associations with the structure and functioning of the brain and PTSD, especially the dysfunction of the cortical regions associated with working memory and sympathetic fight-and-flight response, including the amygdala and PFC, in response to life-threatening cues [13,53,54]. Belkin *et al.* observed that combat veterans with PTSD mount an exaggerated sympathetic response, measured as an increase in the heart rate and blood pressure, when exposed to combat-related triggers [14]. NE was found in excess in this process and has been linked to arousal, attention, and hypervigilance.

Research has shown the effectiveness of α_2_ agonists in inhibiting the PFC-mediated reflexive stress-driven behavior and symptoms associated with NE excess [22,55]. α_2_ agonists directly act on the presynaptic and postsynaptic α_2_ adrenoceptors in the brainstem, thus decreasing the release of NE and strengthening PFC top-down control to resist impulsive actions, improve emotional regulation, and reduce the stress response [56,57]. Despite the limited evidence, α_2_ agonists have been shown to produce a damping effect on the NE activity in CNS [17]. Guanfacine binds specifically to the 2a sub-receptor of the α_2_ receptor, while clonidine acts on 2a, 2b, and 2c sub-receptors, explaining the sedating properties of the latter [14]. Our study found that α_2_ agonists are mostly useful in the management of intrusive and hyperarousal symptoms such as nightmares, aggression, and insomnia, which are credited to excessive noradrenergic activity. The sedating side effect of clonidine may be seen as ‘desirable’ as it helps relax agitating PTSD patients.

Clonidine and guanfacine have shown clinical benefits in treating PFC dysfunction-related symptoms in traumatized or abused children, including impaired self-control, re-experiencing the trauma, avoidance, and hyperarousal [18]. However, the lack of evidence-based studies (clinical trials) and small patient populations in the included studies have limited the scope of α_2_ agonist use in pediatric PTSD. Our systematic review has shown similar results in the improvement of symptoms linked to the autonomic nervous system and cortical dysfunction, such as aggressive self-injurious behavior, mood instability, sleep disturbances, hyperarousal, and executive dysfunction.

Clinical Implications and Future Direction

Though trauma-focused CBT has the most evidence for treating pediatric PTSD, limited access to trauma-focused cognitive behavioral therapy and a lack of TF-CBT-trained psychotherapists curbs its effective utilization around the globe. The lack of a systematic approach to managing PTSD in pediatric patients, the unavailability of effective alternative treatment options, and the lack of evidence-based clinical trials call for more research in the field of psychopharmacotherapy. Extended-release α_2_ agonists are FDA-approved for the treatment of ADHD in children and adolescents, a disorder that has multiple overlapping features with PTSD, such as impulsivity and poor emotional and behavioral regulation. α_2_ agonists have a safety profile suitable for use in ages below 18 years. Thus, double-blinded, randomized controlled trials are needed to find definitive results to form evidence-based guidelines and improve the treatment of PTSD in children and adolescents.

Limitations

One of the major limiting factors in our study is the sample size, with a total of 50 patients from 10 different studies. Another is the lack of evidence-based research. Although treatment of PTSD in children and adolescents with clonidine has been observed to be effective in clinical practice, there are no double-blinded RCTs for α_2_ agonist use in PTSD in the pediatric age group, and open-label studies tend to be affected by various biases, including selection bias, observer bias, and expectancy bias. Third, children and adolescents suffering from PTSD commonly present with overlapping comorbid conditions such as ADHD, anxiety disorders, reactive attachment disorder, and depression. The presence of disorders like ADHD and depression could bias the effect of α_2_ agonists seen on PTSD symptoms like hypervigilance, impulsivity, mood instability, and self-injurious behavior which are also seen in ADHD and depression commonly. The use of medications for these comorbid disorders could impact PTSD symptoms too, thereby affecting the validity of the results. The small number of relevant studies, the limited study population (sample sizes of 50), the different rating scales used, and the limitations in study design all prevent definitive conclusions from being drawn regarding the use of α_2_ agonists for PTSD in children and adolescents.

## Conclusions

In a systematic review of case reports and open-label trials, α_2_ agonists (clonidine and guanfacine) have shown a reasonable rationale for the management of symptoms associated with PTSD in children and adolescents in practice; however, the evidence base is still quite limited. Sedation, fatigue, dry mouth, and dizziness are the common adverse effects. Future double-blinded, randomized, placebo-controlled trials are needed to assess the efficacy and safety of α_2_ agonists.
